# DO OLDER ADULTS WITH HIV HAVE A SOCIAL NETWORK DEFICIT? EVIDENCE FROM AGINCOURT, SOUTH AFRICA

**DOI:** 10.1093/geroni/igz038.627

**Published:** 2019-11-08

**Authors:** Markus H Schafer, Laura Upenieks, Julia DeMaria

**Affiliations:** University of Toronto, Toronto, Ontario, Canada

## Abstract

HIV/AIDS has had a substantial social and economic impact on Sub-Saharan Africa, and research is only beginning to examine the prevalence and consequences of HIV infection among older adults in this region. Though informal social networks provide crucial resources for older people managing health problems, little is known about how the form and function of such networks differs by HIV status. Drawing from theories of health stigma and network mobilization, we use egocentric network data from HAALSI, the Health and Aging in Africa: A Longitudinal Study of an INDEPTH Community in South Africa (N=5,059). HAALSI is a community-based study centered in Agincourt, South Africa, and focuses on adults ≥40 years of age. Approximately 12% of this sample is HIV positive. Results of multivariable logistic and Poisson regression reveal three main findings. First, relative to those without HIV, infected older adults have larger personal networks—including more kin and more non-kin network members. Second, HIV status has no discernible impact on whether people receive regular emotional support from those in their networks. Third, older adults who have disclosed their HIV status have a relatively high proportion of kin members in the close networks relative to those not infected with HIV and to those with HIV who have not disclosed their disease. Our findings point to the need for further research on the implications of social networks for outcomes such as well-being and health care delivery among older AIDS patients in the Global South.

